# Effects of Cr Content on Microstructure and Mechanical Properties of Co-Free FeCr_y_NiAl_0.8_ High-Entropy Alloys

**DOI:** 10.3390/ma16093348

**Published:** 2023-04-25

**Authors:** Puchang Cui, Wei Wang, Zhisheng Nong, Zhonghong Lai, Yong Liu, Jingchuan Zhu

**Affiliations:** 1 School of Materials Science and Engineering, Harbin Institute of Technology, Harbin 150001, China; 2 State Key Laboratory of Solidification Processing, Northwestern Polytechnical University, Xi’an 710072, China; 3 School of Materials Science and Engineering, Shenyang Aerospace University, Shenyang 110136, China; 4 Center for Analysis, Measurement and Computing, Harbin Institute of Technology, Harbin 150001, China; 5 National Key Laboratory for Precision Hot Processing of Metals, Harbin Institute of Technology, Harbin 150001, China

**Keywords:** high-entropy alloys, microstructure, mechanical property, work-hardening behavior

## Abstract

High-entropy alloys have gained widespread concern in response to the increased requirements for future high-temperature structural superalloys. By combining phase-diagram calculations with microhardness, compression behavior measurements at room temperature, and elevated temperature conditions, the very important role of the Cr element on the microstructure and properties is deeply revealed, which provides candidates materials for future high-temperature alloy applications. The increment of Cr favors the regulation of the two-phase fraction and distribution. The thermodynamic calculations illustrate that the density and melting point of the HEAs showed an increasing trend with the increase of the Cr content. The typical worm-like microstructure of the Cr_0.6_ alloy with a dual BCC structure was detected. Meanwhile, on the one hand, the increment of the Cr elements results in a considerable optimization of the mechanical properties of the alloy in terms of strength and ductility at room temperature. The corresponding compressive strength and plasticity of Cr_0.6_ alloy at room temperature are 3524 MPa and 43.3%. On the other hand, the high-temperature mechanical properties of the alloy are greatly enhanced. At 1000 °C, the yield strength of the Cr_0.6_ alloy is about 25 MPa higher than that of the Cr_0.4_ alloy. The superior mechanical properties are attributed to the pronounced work-hardening response, and the work-hardening behavior of Cr-containing HEAs was systematically analyzed by employing the modified Ludwik model. The higher content of Cr helps the resistance of the local deformation response, improving the nonuniform strain and promoting the balance of strength and ductility of the alloys.

## 1. Introduction

Developing lightweight alloys with excellent strength and ductility is always the objective for the application of structural alloys due to the demand for high-performance superalloys in increased harsh working conditions. High-entropy alloys (HEAs) and multiprincipal element alloys have received considerable interest for community experts and have become a worldwide hot spot since Cantor and Yeh reported their first publication [1,2]. Cr-containing HEAs have exhibited promising potential when applied in high-temperature industrial fields as a result of their remarkable strength [3,4,5,6], favorable ductility [7,8,9], distinguished corrosion resistance [10,11,12], superior wear and oxidation resistance [13,14,15], and acceptable thermal stability [16,17].

So far, great endeavors have been devoted to examining the properties of Cr-containing HEAs with the incorporation of the Cr element, not limited to the investigations of corrosion behavior, high-temperature oxidation behavior, and thermal stability of the HEAs, especially regarding the evolution of the microstructure and mechanical behavior with different Cr content, is still a top research priority. The increase of the Cr element promotes the formation of new phases, including the Sigma phase [18,19] and Laves phase [20]. It has been reported that the Cr element acts as a stabilizer for FCC at low contents and affects the formation of the BCC phases at high contents, which promotes the modulation of the mechanical properties of HEAs [21,22,23]. Meanwhile, the increase of the Cr element in HEAs improves the strength and hardness at the expense of the ductility of the alloys to some extent [24,25,26]. It has been reported that the volume fraction of the BCC phase increased as a function of the Cr concentration in FCC + BCC dual-phase Cr_x_MnFeNi HEAs [23]. The microstructure and mechanical properties of (Al_7_Co_24_Cr_21_Fe_24_Ni_24_)_100−x_Cr_x_ HEAs are optimized and the high fracture strength with the value of 2830 MPa and the plastic strain of 24.9% was obtained [24]. Generally, it is assumed that the Cr content plays a decisive role in the enhancement of the corrosion properties of the Cr-containing alloys [27]. The microstructure and the corrosion and oxidation behaviors in Cr-containing HEAs with different Cr contents are also investigated in detail [28,29,30]. The single-phase FCC Ni_38_Fe_20_Cr_6_Mn_18_Co_18_ HEAs containing 6% Cr displayed passivity behavior at a relatively low Cr concentration [31]. Among the numerous HEAs, the Co-free and Cr-containing HEAs demonstrated outstanding property features [32,33,34], and the Co-free HEAs with target microstructures can be obtained by advanced preparation approaches [35]. Recent publications [36,37,38,39] have revealed that the composition manipulations of the Fe-Cr-Ni-Al system alloy determine the mechanical properties, corrosion properties, oxidation properties, damping capacity, and the improvement of preparation techniques influences the alloy properties. Yet, the effect of Cr elements on the microstructure and properties of Cr-containing Fe-Cr-Ni-Al HEAs, and their corresponding mechanisms, need to be further expounded. Thus, an attempt is tried to achieve optimization of mechanical properties through modifying the component concentration to tellingly overcome the mechanical properties trade off and to discuss the potential mechanisms of the effect of the Cr element on the mechanical response of the HEAs.

In this paper, it is expected that Cr-containing HEAs possess distinguished mechanical properties, in conjunction with the strategy of alloying Cr elements, combined with thermodynamic predictions, served as an effective method [40] and the experimental methods, the composition, microstructure, including phase formation, and mechanical properties of the current as-cast alloys are systematically investigated. The present proposed alloys exhibit excellent mechanical properties with high work-hardening capability, which offers a clue for the advancement of high-temperature alloys.

## 2. Materials and Methods

The current prepared FeCr_y_NiAl_0.8_ (y = 0.6, 0.4, 0.2 named as Cr_0.6_, Cr_0.4_, and Cr_0.2_, respectively) HEA specimens were fabricated by vacuum arc-melting approaches. The raw materials of Fe, Cr, Ni, and Al, with purities above 99.9%, were put in the furnace in accordance with the melting points, and the alloy samples were remelted and flipped at least three times to enhance homogeneity. The phase structure identifications of the three alloys were performed by employing Cu radiation X-ray diffraction (XRD) with the scanning angle from 20° to 100°. The morphology and elemental mapping of the as-cast alloys with the dimension of 10 mm × 10 mm × 5 mm were detected by scanning electron microscopy (SEM) and the energy dispersive spectrometer (EDS). The microhardness of the alloys was measured by an MHVD-5AP digital microhardness Vicker’s tester with the parameters of 0.5 kgf load and 15 s. The obtained hardness values with an error bar were determined for every specimen. The low-magnification morphologies of the alloys, including the appearance of indentation, were observed by optical microscopy (OM).

The density, melting point, and constituent phase prediction of the current FeCr_y_NiAl_0.8_ HEAs were calculated by JMatPro software (JMatPro v7.0, State Key Laboratory of Solidification Processing, Northwestern Polytechnical University, Xi’an, China). Compressive mechanical behavior tests were performed utilizing an AG-X Plus 250 kN/50 kN Electronic Universal Testing Machine under compressive rates of 2 mm/min and 0.5 mm/min at room temperature, 800 °C, 900 °C, and 1000 °C, respectively. High-temperature compression tests and room-temperature compression tests adopted the same specimen size with Φ4 mm×6 mm. The equations $\sigma_{T}=\left(1-\varepsilon \right)\sigma$ and $\varepsilon_{T}=\ln\left(1-\varepsilon \right)$ were adopted to convert the engineering stress (σ) and strain (ε) to true stress ($\sigma_{T}$) and strain ($\varepsilon_{T}$) relationships.

## 3. Results and Discussion

### 3.1. Constitutional-Phase Formation and Microstructure Evolution as a Function of Cr Addition

**Figure 1:**
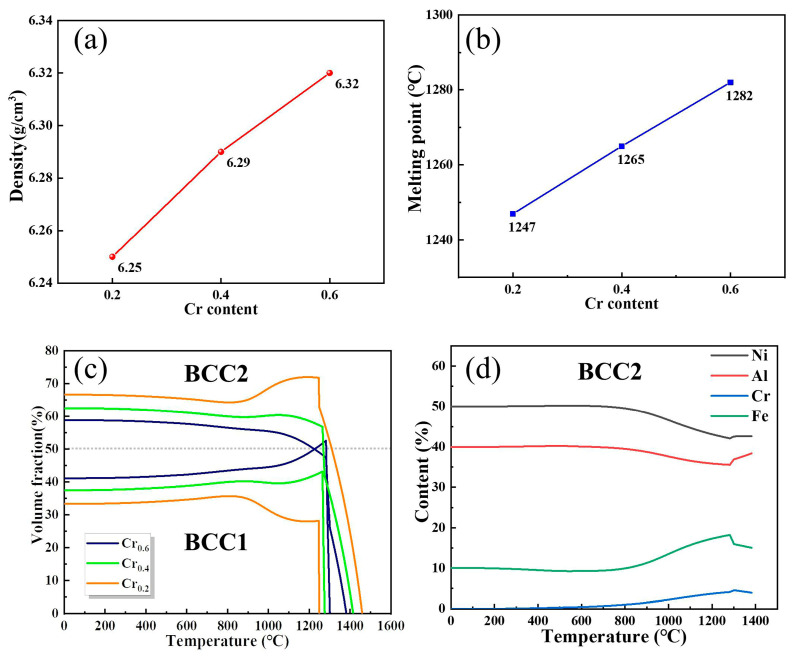
Predicted density, melting point, and constitutional phase of the present investigated HEAs: (**a**,**b**) represents the calculated density and melting point results for the three FeCr_y_NiAl_0.8_ HEAs with varied Cr content, respectively; (**c**) the predicted volume fraction of constitutional phase for proposed Cr_0.6_, Cr_0.4_, and Cr_0.2_ alloys varied with elevated temperature; (**d**) elemental composition in the BCC2 phase for Cr_0.6_ alloy.

In the current work, the three FeNiCr_y_Al_0.8_ system alloys with different Cr content (the subscript y = 0.6, 0.4, 0.2) were designed to investigate the effect of Cr concentration on the formation of alloy microstructure, density, and melting point to provide essential research for the application of the alloys in high-temperature fields. First, the melting points and densities of the alloys were predicted and the results are shown in Figure 1a,b. It can be observed that the density of the alloy presents a trend of increasing and the melting point is also on a trend of increasing with the increment of Cr content. From the predicted values, the density of the current alloys with varied Cr elements ranges between 6.25–6.32 g/cm^3^, and the melting point is in the range of 1247–1282 °C. Compared to the Inconel 718 superalloy with a density of 8.19 g/cm^3^, the density values of all the designed compositions of the alloys were less than 7 g/cm^3^, which is beneficial for lightweight superalloys with higher service temperatures. Thus, Cr_0.6_ HEA has great potential to become an alternative material to meet the requirements of lightweight development in the aviation and aerospace industries. Figure 1c,d plots the fraction of phase in alloys containing different Cr contents and the elemental composition of the BCC2 phase in the Cr_0.6_ alloy as a function of temperature. It is obvious that the variation of the two-phase content in the alloy increases with the decrease of the Cr content, and the most significant difference between the BCC1 and BCC2 phases in the Cr_0.2_ alloy indicates that the decrease of the Cr content aggravates the formation of dual phase segregation, which is not conducive to the coordination of the deformation of the alloy. Meanwhile, the Cr_0.6_ HEA shows a slight difference in the content of the two phases in the low-temperature range and the content of the BCC2 phase is higher than that of the BCC1 phase. In addition, it can be noticed that the content of the BCC2 phase tends to increase as the Cr content decreases, which is speculated that the Cr element is the stabilizing element in the BCC1 phase. Further investigation of the elemental composition of the BCC2 phase in the Cr_0.6_ alloy suggests that the BCC2 phase is enriched with Ni and Al elements, and the difference in the Fe-Cr elemental content of the alloy tends to become larger, while the difference in the Ni-Al elemental content remains steady alterations. Combined with the predicted results, no phase transformation behavior was discovered at temperatures below 1000 °C, and only a slight change in the two-phase content was observed, indicating that the alloy is more suitable for high-temperature service environments due to its excellent thermal stability compared to conventional alloys.

**Figure 2:**
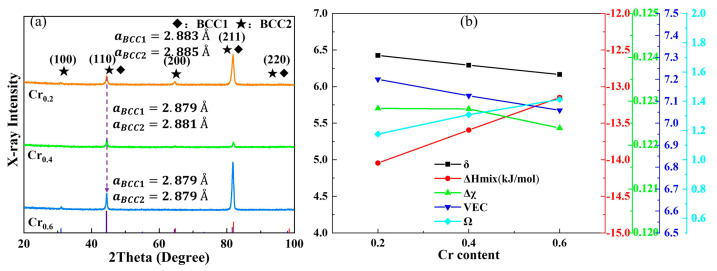
Phase formation analysis of the current HEAs: (**a**) the XRD patterns for proposed Cr_0.6_, Cr_0.4_, and Cr_0.2_ alloys with a diffraction angle range from 20 to 100 degrees, and the diffraction peak information of the reference standard phases are presented at the bottom of Figure 2a; (**b**) displays the empirical descriptions modeling results of the investigated FeCr_y_NiAl_0.8_ HEAs.

Figure 2a depicts the XRD results of the Cr_0.6_, Cr_0.4,_ and Cr_0.2_ HEAs and the phase structures of the alloys are determined. The obtained results show that these alloys consist of a disordered BCC phase (marked by a black diamond) and an ordered B2 phase (marked by a five-pointed star). These are in agreement with the results reported for the Fe-Cr-Ni-Al alloys [41,42]. A decreasing tendency of the lattice constant is displayed, which is validated by the right shift of the diffraction peak with an increment of Cr concentrations. It can be confirmed that the lattice constants of the two phases gradually decrease with the increase of Cr content, and the lattice constants of the BCC1 and BCC2 phases varied from 2.883 Å to 2.879 Å and 2.885 Å to 2.879 Å, respectively. Combined with the phase-analysis results, several empirical descriptors, including mixing enthalpy ($\Delta H_{\mathrm{mix}}$) extrapolated from the binary mixing enthalpies, valence electron concentration (VEC), atomic-size misfit (δ), phase-stability parameter (Ω), and electronegativity difference (Δχ), that vary with the Cr content, were calculated as presented in Figure 2b. These parameters for HEAs have been extensively derived to use the predictions of the phase structural analysis of HEAs [43,44,45,46]. Previous investigations have developed the empirical rules of solution phase precipitation, and the corresponding criteria are satisfied: −15 kJ/mol $\leq \Delta H_{\mathrm{mix}}\;\leq$ 5 kJ/mol, δ ≤ 6.6, and Ω ≥ 1.1. Meanwhile, the VEC values of 6.87 and 8 are proposed to be the threshold of the BCC and FCC phase formation. The equations are as follows (1)–(7):

(1) $$\Delta H_{\mathrm{mix}}=\sum_{i=1,i\neq j}^{n}4\Delta H_{\mathrm{AB}}^{\mathrm{mix}}c_{i}c_{j}$$

(2) $$\delta =\sqrt{\sum_{i=1}^{n}c_{i}{\left(1-\frac{r_{i}}{\bar{r}}\right)}^{2},}\;\bar{r}=\sum_{i=1}^{n}c_{i}r_{i}$$

(3) $$\mathrm{VEC}=\sum_{i=1}^{n}c_{i}{\left(\mathrm{VEC}\right)}_{i}$$

(4) $$\Omega =\frac{T_{m}\Delta S_{\mathrm{mix}}}{\left|\Delta H_{\mathrm{mix}}\right|}$$

(5) $$T_{m}=\sum_{i=1}^{n}c_{i}{\left(T_{m}\right)}_{i}$$

(6) $$\Delta S_{\mathrm{mix}}=\sum_{i=1}^{n}\left({-\mathrm{Rc}}_{i}\ln\left(c_{i}\right)\right)$$

(7) $$\Delta \chi =\sqrt{\sum_{i=1}^{n}c_{i}{\left(\chi_{i}-\bar{\chi }\right)}^{2}},\;\bar{\chi }=\sum_{i=1}^{n}c_{i}\chi_{i}$$

where $c_{i}$ is the atomic content of ith type atoms; $\Delta H_{\mathrm{AB}}^{\mathrm{mix}}$ represents binary mixing enthalpy; $r_{i}$ refers to the radius of ith type atoms; ${\left(\mathrm{VEC}\right)}_{i}$ is the VEC of ith type atoms; $T_{m}$ means melting point; $\Delta S_{\mathrm{mix}}$ means mixing entropy; R is the gas constant; $\chi_{i}$ and $\bar{\chi }$ correspond to electronegativity and average electronegativity, respectively. Therefore, the calculations of the phase-structure formation of the current HEAs for nominal composition are provided as shown in Figure 2b. It can be found that the mixing enthalpy of the alloy increases with the increment of Cr content. However, the negative mixing enthalpy favors the formation of stable solid-solution structures. The atomic-size difference presents a gradual decrease with the increase of Cr content, which is related to the small variation of the atomic size of the Cr element from Fe and Ni atoms in the current alloy. With the increment of Cr elements, the atomic sizes are all less than 6.6, which indicates that the current HEAs form a stable solid solution structure. At the same time, it can be identified that the phase stability parameters of the alloys are all higher than 1.1, indicating that the alloys possess high stability characteristics. However, the current VEC values are situated in the FCC + BCC two-phase zones according to the empirical method and exhibit a decreasing trend from 7.2 to 7 with increasing Cr content, indicating that the current experimental results do not meet the empirical relationship and, therefore, further correction efforts are necessary.

**Figure 3:**
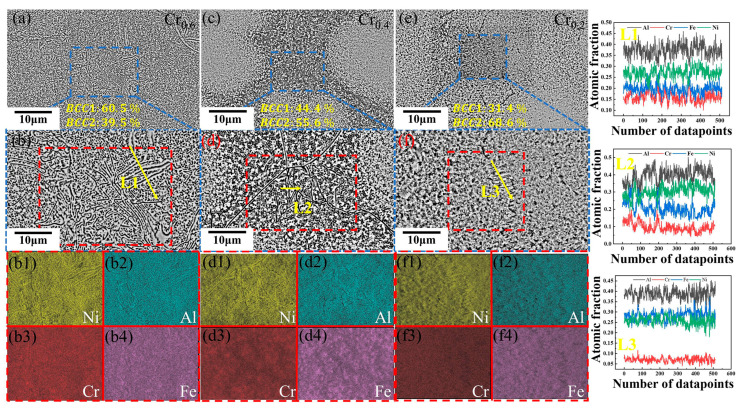
Microstructure morphologies and elemental distribution results of the current FeCr_y_NiAl_0.8_ HEAs: (**a**,**b**) shows the microstructure features of the Cr_0.6_ alloy, The corresponding elemental mappings of Ni, Al, Cr, and Fe are displayed in (**b1**–**b4**). (**c**,**d**) and (**e**,**f**) represent the selected SEM images for Cr_0.4_ and Cr_0.2_ specimens, respectively. The corresponding elemental mappings of Ni, Al, Cr, and Fe of Cr_0.4_ and Cr_0.2_ specimens are displayed in (**d1**–**d4**) and (**f1**–**f4**). The line scanning profiles of L1 to L3 in three alloys marked by the yellow arrow is displayed on the right region, demonstrating the existence of the dual-phase microstructures.

The SEM microstructures with bright and dark gray areas of the three as-cast alloys Cr_0.6_, Cr_0.4_, and Cr_0.2_ are shown in Figure 3. From Figure 3a–e, it is evident that grain boundaries and typical dual phases are observed. In general, the Cr is found to enhance the formation of sigma phases in the HEAs. The contents of the BCC1 phase in the investigated alloys are calculated and analyzed increasing from 31.4% in Cr_0.2_ alloy to 60.5% in Cr_0.6_ alloy with the increase of Cr content, which is consistent with the formation of the BCC1 phase associated with the increase of Cr elements and thermodynamic predictions. The increase in the BCC1 phase content caused by the increase in Cr elements can be attributed to the Cr element acting as the stabilizing elements for Fe-Cr-rich phases in high-entropy alloys, and the preferential formation of the BCC2 phase enriched NiAl element (more negative mixing enthalpy in thermodynamic aspects) has an influence on the content of the BCC1 phase. Meanwhile, the spinodal decomposition features of the current alloys are presented. The elemental mappings of the dual phase Cr_0.6_ alloy are measured by the EDS results of microregions and line scanning, as shown in Figure 3(b1–b4) and line L1. Meanwhile, the selected regions in the Cr_0.4_ and Cr_0.2_ alloys marked by blue and red dotted boxes are displayed in Figure 3c–f. Slight segregation for the Ni-Al rich phase in the four constituent elements was detected and the dropping of the actual Cr content in the three alloys is presented with a decrement of Cr concentration (mapping and line profiles of L1–L3), which is consistent with the results from the previous dual-phase Fe-Cr-Ni-Al alloy system [47,48]. With increasing Cr content, it is easier to form a dual-phase woven modulated microstructure that is favorable to enhancing the mechanical properties of the alloys.

### 3.2. Influence of Cr on Mechanical Properties of FeNiCr_y_Al_0.8_ HEAs

#### 3.2.1. Microhardness Variation with Different Cr Content

**Figure 4:**
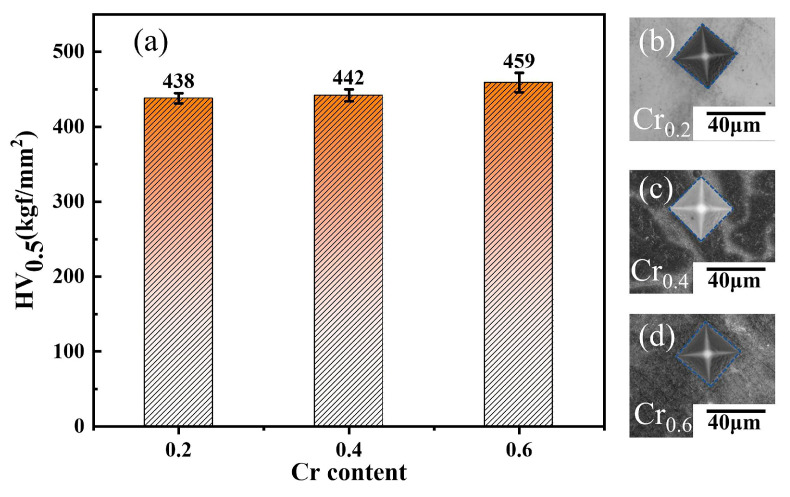
Variation of measured microhardness and the appearance of indentation with different Cr content in FeCr_y_NiAl_0.8_ HEAs: (**a**) microhardness values; (**b**–**d**) are corresponding to the representative morphology of indentation of present alloys.

Figure 4 plots the Vickers microhardness results of three fabricated alloys, it can be noticed that the hardness value of the as-cast alloys presents a decreasing trend with the reduction of the Cr element. However, the steady variation range of decrease is presented, and the hardness value decreases from HV459 of the Cr_0.6_ specimen to HV438 of the Cr_0.2_ specimen, approximately a decrease of HV21, which is associated with the reduction of Cr content in the present alloy to the decrease of strength of the alloy. To some extent, the increased content of the BCC1 phase promoted by the increment of Cr concentration could also promote the enhancement of hardness and mechanical properties [49]. The hardness results indicate that the Cr_0.6_ alloy presents more distinguished mechanical properties by adding the Cr elements strategy. Meanwhile, the representative appearance of indentation of the three alloys is shown in Figure 4b–d.

#### 3.2.2. Room-Temperature Mechanical Behavior of FeCr_y_NiAl_0.8_ HEAs with Different Cr Concentration

**Figure 5:**
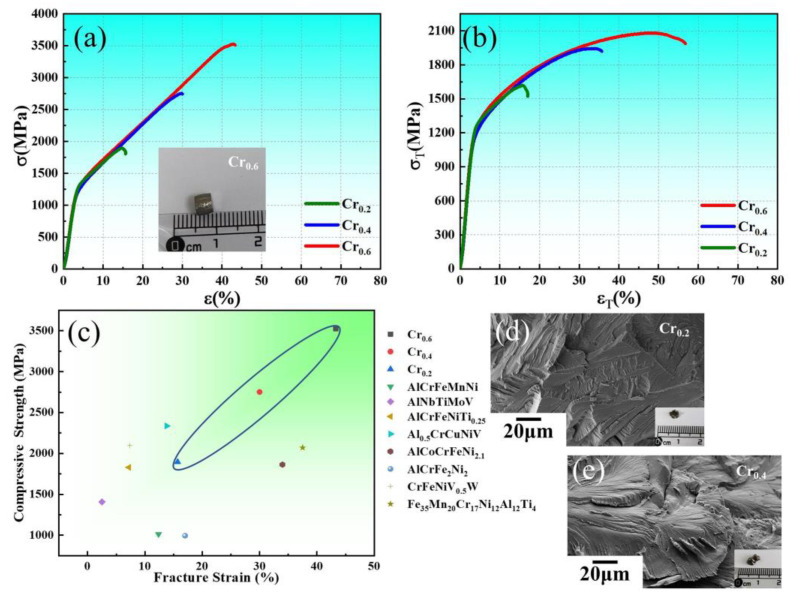
Compressive stress versus strain response under room temperature for the developed three HEAs: (**a**) engineering compressive curves for Cr_0.6_, Cr_0.4_, and Cr_0.2_ alloys and the macroscopic fractured photograph of Cr_0.6_ alloy after the measurement is illustrated in the inset image of Figure 5a; (**b**) true compressive relationships for Cr_0.6_, Cr_0.4_, and Cr_0.2_ alloys; (**c**) comparison of compressive strength and fracture for Cr_0.6_, Cr_0.4_, and Cr_0.2_ alloys with representative developed alloys [37,50]; SEM images and macroscopic appearance (insert illustration) of the fractured surface after compressive testing of the (**d**) Cr_0.2_ alloy and (**e**) Cr_0.4_ alloy.

Figure 5 displays the room-temperature compressive stress versus strain curves of the three prepared alloys, and Figure 5a,b exhibit the engineering stress–strain curves and the true stress–strain curves for the different Cr contents, respectively. In the currently investigated alloys, it can be observed that the overall strength and plasticity of HEAs are substantially increased with the increase of Cr elements, and the corresponding mechanical property parameters are tabulated in Table 1. A similar elastic deformation stage and yield strength values are observed in three alloys. Fascinatingly, the compressive strength of the Cr_0.6_ alloy is revealed to be the largest one, about 3524 MPa, and the compressive strain is about 43.3%. As the Cr content reduces from Cr_0.6_ alloy to Cr_0.4_ alloy, the strength and ductility of the specimen decrease with a compressive strength of 2751 MPa and a compressive strain of about 30% in the Cr_0.4_ alloy. From the macroscopic fractured image of the Cr_0.6_ alloy shown in the inset illustration of Figure 5a, it can be observed that there was no complete fracture of the Cr_0.6_ alloy and macrocracks were observed along approximately 45 degrees (shear fracture), indicating that the Cr_0.6_ alloy exhibits a favorable strength–ductility combination. However, the excellent work-hardening ability of the two alloys is observed, ensuring the uniform deformation of HEAs to achieve large ductility. In contrast, the Cr_0.2_ alloy displays a compressive strength of 1896 MPa and a compressive strain of only 15.7%. To estimate the comprehensive behavior of current HEAs, several developed HEAs retain limited mechanical properties [37,50]. Therefore, the strength and ductility of the Cr_0.6_ alloy are well balanced. With a long work-hardening stage, the Cr_0.6_ alloy can effectively optimize the mechanical properties of the alloys. Additionally, the fracture surfaces of the Cr_0.4_ and Cr_0.2_ alloys were carried out to investigate their fracture types. Further, the fracture-surfaces results of both alloys reveal that the fracture characteristics of the alloys present typical river-like patterns with cleavage steps, which indicate that the main fracture mode in both alloys corresponds to cleavage brittle fracture as shown in Figure 5d,e.

#### 3.2.3. High-Temperature Mechanical Behavior of FeCr_y_NiAl_0.8_ HEAs with Different Cr Content

**Figure 6:**
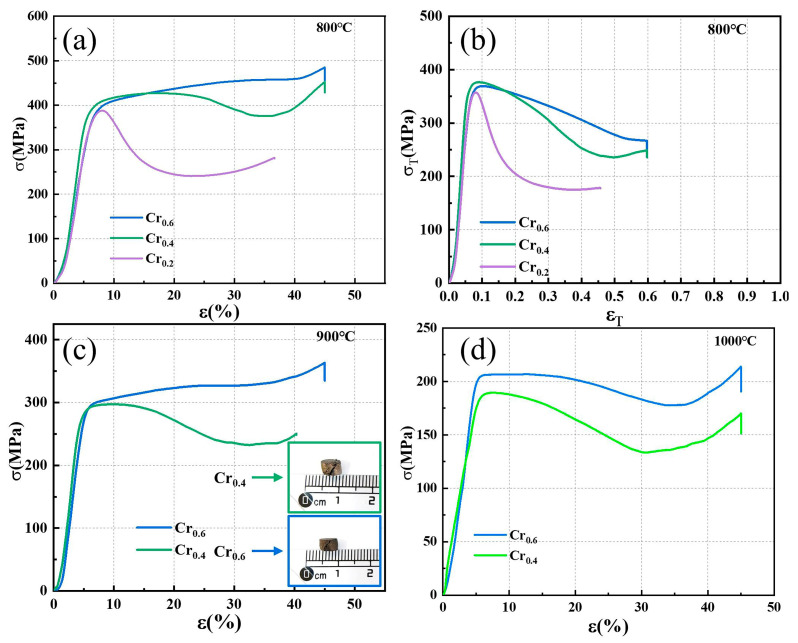
Compressive response under elevated temperature for developed HEAs: (**a**,**b**) represent the engineering and true compressive behavior at 800 °C for Cr_0.6_, Cr_0.4_, and Cr_0.2_ alloys, respectively. (**c**,**d**) exhibit the engineering compressive behavior at 900 °C and 1000 °C for Cr_0.6_ and Cr_0.4_ alloys, respectively. The insert images represent the macroscopic appearance of Cr_0.6_ and Cr_0.4_ fractured specimens after measurement.

To further investigate the high-temperature mechanical properties of the alloys to meet the background of high-temperature environment applications, the three as-cast alloys were subjected to high-temperature compression measurements at 800 °C, 900 °C, and 1000 °C. Figure 6a,b shows the engineering and true stress versus strain curves of the three HEAs at 800 °C. The three alloys possess a high strength of about 400 MPa at 800 °C. It can be noticed that continuous strengthening is observed after the yielding and softening effects of the three alloys can be seen from the true stress and strain behaviors which contribute to the recovery and the recrystallization effects are superior to the effect of work hardening. It can be observed that the Cr_0.6_ alloy has a similar yield strength to the other alloys at 800 °C, though the Cr_0.6_ alloy is more resistant to high-temperature deformation behavior with an increasing applied strain and does not soften easily. Meanwhile, the Cr_0.6_ alloy exhibits significant resistance to softening compared to the Cr_0.4_ alloy until the temperature increases to 1000 °C as shown in Figure 6c,d, and the yield strength has a more moderate increment than the Cr_0.4_ alloy with the values of 25 MPa.

#### 3.2.4. Effect of Cr Content on Work-Hardening Behavior of FeNiCr_y_Al_0.8_ Alloys

Generally, the high work-hardening behavior in the alloy is reflected by favorable local plastic deformation resistance, contributing to the alloy with the excellent strength–ductility combination. Thus, it is necessary to investigate the work-hardening behavior of the current HEAs. Based on the previous results of room-temperature compression, the current HEAs exhibit excellent work-hardening ability. Furthermore, the key parameter of the work-hardening effect is the work-hardening exponent n. Meanwhile, a higher value of n expresses the resistance to uniform deformation, endowing acceptable cold formability for the studied HEAs.

A large number of mathematical relationships have been proposed to describe the work-hardening behavior based on the work-hardening interpretation from previous studies. The most general expression proposed by Hollomon [51,52] has been displayed:

(8) $$\sigma_{T}=K\varepsilon_{T}{}^{n}$$

where *K* is associated with the strength coefficient, which is a constant, and *n* means the work-hardening exponent.

Ludwik has introduced an additional term to further explain the nonzero stress influence stem from the Hollomon equation, as expressed in Equation (9) [53,54]:

(9) $$\sigma_{T}=\sigma_{0}^{T}+K\varepsilon_{T}{}^{n}$$

In the present work, since the Hollomon model is quite simple, the modified Ludwik expression was chosen, which can be described as Equation (10) [55]:

(10) $$\sigma_{T}=k\varepsilon_{T}{}^{n_{1}+n_{2}ln\varepsilon_{T}}$$

Here, the factor $\varepsilon^{n_{2}ln\varepsilon }$ indicates that $k\varepsilon^{n_{1}}$ is improved by $\varepsilon^{n_{2}ln\varepsilon }$.

The data of the fitting results are summarized in Table 2. It can be noted that the current adopted modified Ludwik model of working-hard behavior exhibits a good fitting effect with high precision (R^2^ > 99%). Thus, the corresponding mathematical expressions after fitting the Cr_0.6_, Cr_0.4,_ and Cr_0.2_ alloys are shown in Equations (11)–(13). Meanwhile, the increment of Cr content can significantly enhance the work-hardening ability of the current HEAs, ensuring an alloy with remarkable ductility characteristics.

(11) $$\sigma_{T\;}={2995.5\varepsilon_{T}}^{0.308+0.012\ln\varepsilon_{T}}$$

(12) $$\sigma_{T}={3124.1\varepsilon_{T}}^{0.343+0.014\ln\varepsilon_{T}}$$

(13) $$\sigma_{T}={2396.7\varepsilon_{T}}^{0.184-0.005\ln\varepsilon_{T}}$$

Based on the above-obtained microstructure and property results, the mechanisms of the Cr content on the microstructure and properties of the current HEAs require further discussion. This investigation of room-temperature and high-temperature mechanical properties of the current HEAs, accompanied by distinct Cr contents, exhibits that the increment of Cr content could promote the room-temperature mechanical properties of the alloy to be significantly enhanced depending on the significant work-hardening effect. Not only that, the optimizations of high-temperature mechanical properties for the current alloys with the increment of Cr contents are also achieved. In general, the local plastic deformation determines the mechanical properties of the alloys to the close-yield strength process. The alloys are susceptible to local deformation that promotes early macroscopic nonuniform deformation, which manifests as a result of inferior plasticity leading to premature failure. Combined with the extracted room-temperature yield-strength results, the three alloys have similar yield strengths, indicating that the increment of the Cr element generates a certain limited solid-solution strengthening effect. The results of the phase diagram reflect that the increment of Cr alters the content and distribution of the constituent phases in the alloy. The increase of Cr content increases the content of the BCC1 phase and decreases the content of the BCC2 phase, which can improve the mechanical properties of the Cr_0.6_ alloy by a heterogeneous dual phase formation. The heterogeneous dual phase deformation contributes to the improvement of the local deformation properties and the coordinated deformation between the weak and strong phases. Further analysis reveals that the increase of the Cr element can make the alloy resist local deformation and prolong the nonuniform plastic-deformation phase, which is realized as an outstanding work-hardening effect at the macroscopic level, endowing the alloy with excellent comprehensive strength and ductility [56,57].

## 4. Conclusions

In this work, the effect on the microstructure and mechanical properties of FeNiCr_y_Al_0.8_ HEA are systematically investigated by adopting the Cr alloying strategy. The main conclusions are as follows:

(1) The thermodynamic prediction results illustrate that the density and melting point of the alloys displayed an increasing trend with the increase of the Cr element. Simultaneously, it can be found that the BCC2 phase content was higher than the BCC1 phase content, and the variation in the dual phase fraction gradually decreased with the increment of the Cr elements. The typical worm-like microstructure of the Cr_0.6_ alloy with dual BCC structures was detected. The higher content of Cr facilitated the regulation of the phase fraction and mapping.
(2) In terms of mechanical properties of the current HEAs, the microhardness of the alloy tended to increase slightly with the increased Cr elements, and the corresponding Vicker’s microhardness of the Cr_0.6_ alloy was HV459. The introduction of the Cr element made the strength and ductility of the mechanical properties of the alloys at room temperature substantially optimized. The compressive strength and plasticity of the Cr_0.6_ alloy at room temperature were 3524 MPa and 43.3%, respectively.
(3) The introduction of the Cr element also significantly improved the high-temperature mechanical properties of the alloy. The strength and plasticity of the Cr_0.6_ alloy were better than those of the Cr_0.4_ alloy and Cr_0.2_ alloy. The yield strength of the Cr_0.6_ alloy was approximately 25 MPa higher than that of the Cr_0.4_ alloy at 1000 °C.
(4) The superior mechanical properties resulted from the obvious work-hardening behavior. The work-hardening behavior of the HEAs was systematically analyzed by adopting the modified Ludwik model. The Cr element improved the local deformation characteristics, and the coordinated deformation of the dual phase was carried out, improving the nonuniform strain and promoting the strength and ductility balance of the alloys.

## Figures and Tables

**Table 1:** Extracted mechanical property indicators varied with Cr content under room-temperature conditions.

Sample	E (MPa)	$\sigma_{s}\;(\mathrm{MPa})$	$\sigma_{b}\;(\mathrm{MPa})$	$\varepsilon_{f}\;(\%)$
Cr_0.6_	3623	1118	3524	43.3
Cr_0.4_	3905	1036	2751	30.0
Cr_0.2_	3642	1103	1896	15.7

**Table 2:** Summarized key parameters fitting results are obtained from the compressive curves in the ambient environment.

Sample	k	$n_{1}$	$n_{2}$	R^2^
Cr_0.6_	2995.5	0.308	0.012	0.99945
Cr_0.4_	3124.1	0.343	0.014	0.99891
Cr_0.2_	2396.7	0.184	−0.005	0.99472

## Data Availability

The data presented in this study are available on request from the corresponding author.
